# Influence of Ethylene Glycol Methacrylate to the Hydration and Transition Behaviors of Thermo-Responsive Interpenetrating Polymeric Network Hydrogels

**DOI:** 10.3390/polym10020128

**Published:** 2018-01-26

**Authors:** Bing Li, Qi Zhong, Dapeng Li, Ke Xu, Lu Zhang, Jiping Wang

**Affiliations:** 1 Key Laboratory of Advanced Textile Materials & Manufacturing Technology, Ministry of Education; Engineering Research Center for Eco-Dyeing & Finishing of Textiles, Ministry of Education; National Base for International Science and Technology Cooperation in Textiles and Consumer-Goods Chemistry, Zhejiang Sci-Tech University, Hangzhou 310018, China; lemon_libing@163.com (B.L.); qi.zhong@zstu.edu.cn (Q.Z.); xk49677559@163.com (K.X.); lunaticzzzzzz@163.com (L.Z.); 2 Silk Institute, College of Materials and Textiles, Zhejiang Sci-Tech University, Hangzhou 310018, China; 3 Department of Bioengineering, University of Massachusetts Dartmouth, North Dartmouth, MA 02747, USA; dli@umassd.edu

**Keywords:** thermo-responsive, interpenetrating polymeric network hydrogel, ethylene glycol methacrylate, *N*-isopropylacrylamide, cotton fabrics

## Abstract

The influence of ethylene glycol methacrylate (EGMA) to the hydration and transition behaviors of thermo-responsive interpenetrating polymeric network (IPN) hydrogels containing sodium alginate, *N*-isopropylacrylamide (NIPAAm), and EGMA were investigated. The molar ratios of NIPAAm and EGMA were varied from 20:0 to 19.5:0.5 and 18.5:1.5 in the thermo-responsive alginate-Ca^2+^/P(NIPAAm-*co*-EGMA) IPN hydrogels. Due to the more hydrophilicity and high flexibility of EGMA, the IPN hydrogels exhibited higher lower critical solution temperature (LCST) and lower glass transition temperature (*T*_g_) when the ratio of EGMA increases. The swelling/deswelling kinetics of the IPN hydrogels could be controlled by adjusting the NIPAAm/EGMA molar ratio. A faster water uptake rate and a slower water loss rate could be realized by increase the amount of EGMA in the IPN hydrogel (the shrinking rate constant was decreased from 0.01207 to 0.01195 and 0.01055 with the changing of NIPAAm/EGMA ratio from 20:0, 19.5:0.5 to 18.5:1.5). By using 2-Isopropylthioxanthone (ITX) as a photo initiator, the obtained alginate-Ca^2+^/P(NIPAAm-*co*-EGMA_360_) IPN hydrogels were successfully immobilized on cotton fabrics. The surface and cross section of the hydrogel were probed by scanning electron microscopy (SEM). They all exhibited a porous structure, and the pore size was increased with the amount of EGMA. Moreover, the LCST values of the fabric-grafted hydrogels were close to those of the pure IPN hydrogels. Their thermal sensitivity remained unchanged. The cotton fabrics grafted with hydrogel turned out to be much softer with the continuous increase of EGMA amount. Therefore, compared with alginate-Ca^2+^/PNIPAAm hydrogel, alginate-Ca^2+^/P(NIPAAm-*co*-EGMA_360_) hydrogel is a more promising candidate for wound dressing in the field of biomedical textile.

## 1. Introduction

Hydrogels are three-dimensional polymeric networks with hydrophilic groups that can absorb from 10–20% up to hundreds of times their dry weight in water [1,2]. Over the past few decades, environment-sensitive hydrogels have attracted a great deal of attention because the materials experience a volume phase transition in response to environmental stimuli such as temperature [3,4], pH [5], solvent composition [6], magnetic field [7], and electric field [8], etc. Among all the stimuli, temperature is the easiest triggering signal for phase transition in hydrogels because it is the most available biomedical index, which can be easily adjusted and controlled. Among these thermal-responsive hydrogels, poly(*N*-isopropylacrylamide) (PNIPAAm) gel is a typical thermosensitive hydrogel that exhibits an abrupt volume transition in response to change of temperature around 33 °C. Its characteristic lower critical solution temperature (LCST) is close to human body temperature [9,10]. LCST was regarded as the threshold value of the hydrogel phase transition. PNIPAAm remains hydrophilic in an aqueous solution with extended polymer chains when the external temperature is below the LCST. Upon heating up to its LCST, PNIPAAm becomes hydrophobic as a result of the separation of polymer chains from water. Because of this unique property, plenty of investigations about the PNIPAAm hydrogels are focused on the phase transition, the effects influencing the transition behavior, and variation of its transition behavior [10]. In addition, PNIPAAm has been extensively investigated in fields of drug delivery [11], tissue engineering [12], cell attachment [13], protein absorption [14], etc. However, one of the most critical shortcomings of traditional PNIPAAm hydrogels is their slow response rate to external temperature change due to the initial formation of a dense skin layer, which delays the diffusion of interior water molecules during the collapse process at temperatures above the LCST [15].

Sodium alginate (SA) is a well-known natural polysaccharide extracted from brown seaweed, which is a promising candidate to obtain hydrogels due to its properties of abundant, biocompatibility, biodegradability, renewable, non-toxic and a relatively low cost [16]. Combining SA and NIPAAm to form interpenetrating polymeric network (IPN) is an effective way of developing IPN-based hydrogel systems, which could accelerate the shrinkage rate because of the water-releasing channels provided by the hydrophilic SA component [17,18,19]. There are a number of reports referring to SA in combination with PNIPAAm. Shi J. et al. [20] synthesized a series of IPN hydrogel beads composed of alginate-Ca^2+^ and poly(*N*-isopropylacrylamide) and investigated the temperature- and pH-modulated equilibrium swelling and drug release with indomethacin. Zhang G. et al. [21] presented the influence of SA/NIPAAm ratio on deswelling of SA/PNIPAAm semi-IPN hydrogels in response to temperature and pH changes. Dumitriu R.P. et al. [22] presented the influence of the crosslinking degree of alginate/NIPAAm content on swelling and mechanical properties and assessed drug release formulation by the theophylline release test of pH 2.2 and pH 7.4 at 37 °C, etc. However, PNIPAAm exhibits high stiffness due to its high glass transition temperature (*T*_g_) [23], which would reduce the comfort when applied in textiles in our present investigation.

Besides the acrylamide hydrogels, another class of thermo-responsive polymers based on poly(methacrylate)s has arose the interests of researchers [24,25,26]. Compared to PNIPAAm, these polymers have a lower *T*_g_ (blow 0 °C); moreover, the LCST of poly(methacrylate)s can be easily adjusted between 5–90 °C by changing the number of ethoxy groups in the side chains [27]. Hence, it is a promising candidate to overcome the drawback of PNIPAAm. By introducing of ethylene glycol methacrylate (EGMA) into alginate-Ca^2+^/PNIPAAm IPN hydrogel, the alginate-Ca^2+^/P(NIPAAm-*co*-EGMA) IPN hydrogels obtained presented the reduced *T*_g_ and provided additional hydroxyl groups to enhance the swelling capability. Hence, the modified hydrogels will be suitable for the applications in the field of textiles, such as the wound dressing.

In this paper, the thermo-responsive hydrogel alginate-Ca^2+^/P(NIPAAm-*co*-EGMA_360_) was synthesized via free-radical polymerization. Thermogravimetry analysis (TGA), differential scanning calorimetry (DSC), and swelling/deswelling ratio were applied to characterize the thermal behaviors of IPN hydrogels, and in order to compare these thermal behaviors, IPN hydrogels were prepared by different NIPAAm/EGMA ratios. Afterward, they were grafted with the cotton fabric by UV photo-grafting. The surface and cross section morphology, temperature-dependent swelling behavior and hand evaluation were discussed to evaluate the cotton fabric grafted hydrogels. Finally, a conclusion was given to summarize the variation of the thermal behaviors of the IPN hydrogels and the hand feeling of cotton-fabric composites.

## 2. Materials and Methods 

### 2.1. Materials

A twill cotton fabric (with a warp and weft density of 122 roll/in. and 85 roll/in., respectively) was supplied by Zhejiang Furun Co., Ltd. (Shaoxing, China). Alginic acid sodium (SA), extracted from brown algae (viscosity ≥ 2000cP, 2% *w*/*v* water solution at 25 °C), and ethylene glycol methacrylate (EGMA, average M_n_ = 360) (EGMA_360_) was purchased from Sigma-Aldrich Co., Ltd. (St. Louis, MO, USA). Ammonium persulfate (APS), *N*,*N*′-Methylenebisacrylamide (MBAA), *N*-isopropylacrylamide (NIPAAm), calcium chloride (CaCl_2_), *N*,*N*,*N*′,*N*′-Tetramethylethylenediamine (TEMED) and 2-Isopropylthioxanthone (ITX) were purchased from Aladdin Chemistry Co., Ltd. (Shanghai, China). NIPAAm was recrystallized from hexane and MBAA was purified by recrystallization from acetone before use. All other chemicals were used as received unless otherwise stated. Deionized water was used throughout.

### 2.2. Preparation of Hydrogels

**Figure 1:**
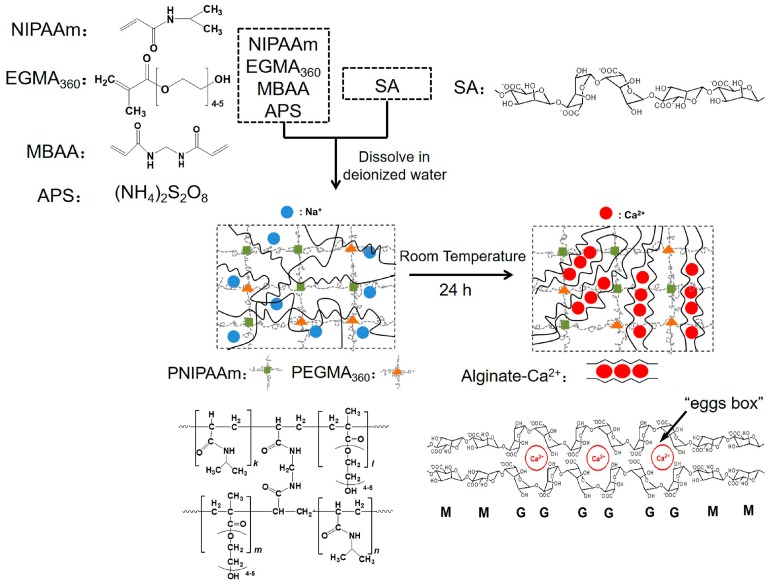
Schematic illustration of the preparation of hydrogels (M represents (1-4)-linked β-d-mmannuronic acid monomer and G represents α-l-guluronic acid monomer).

A series of IPN hydrogels were prepared via free radical polymerization with APS as redox initiator, MBAA as chemical cross-linker, and CaCl_2_ as ionic cross-linker. Briefly, SA, and MBAA, NIPAAm or NIPAAm /EGMA_360_ were carefully dissolved in 20 mL deionized water under constant stirring of 250 rpm to obtain a homogeneous and transparent solution. The solution was then transferred into a tube and degassed with nitrogen for 30 min. The polymerization was initiated by adding 20 mg APS and 6 μL TEMED to the solution, followed by 12-h curing at room temperature to complete. The preparation temperature below and above LCST will significantly influence the properties of NIPAAm-based hydrogels. To precisely control the reaction process and avoid phase separation of PNIPAAm, the temperature is controlled below LCST (34 °C) during the hydrogel preparation. The resulting PNIPAAm hydrogel was then immersed in 1% (*w*/*v*) CaCl_2_ aqueous solution at room temperature for 24 h to form the alginate-Ca^2+^/PNIPAAm IPN hydrogel or alginate-Ca^2+^/P(NIPAAm-*co*-EGMA_360_) IPN hydrogel. Although there are doubts about PNIPAAm and its toxicity and biocompatibility to the human body, the obtained hydrogel in our investigation will be used as wound dressing. Hence, it did not directly go into the human body, and the toxicity of hydrogel can be neglected. To remove the residual monomer, initiator, and cross-linkers in the hydrogel, the obtained hydrogels or fabric grafted hydrogels were placed in distilled water, and the distilled water was exchanged every few hours to extract residual or unreacted chemicals. The reaction mechanism is presented in Figure 1. The IPN samples prepared under various conditions for study are listed in Table 1.

### 2.3. Preparation of Cotton Fabric Grafted Hydrogels

**Figure 2:**
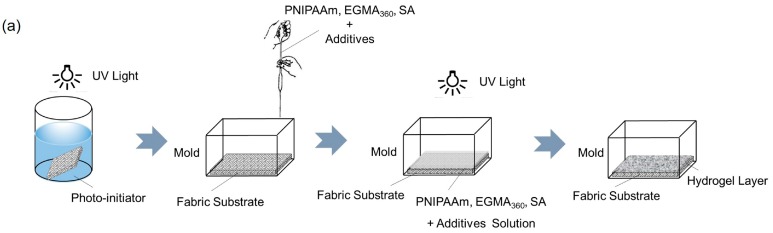

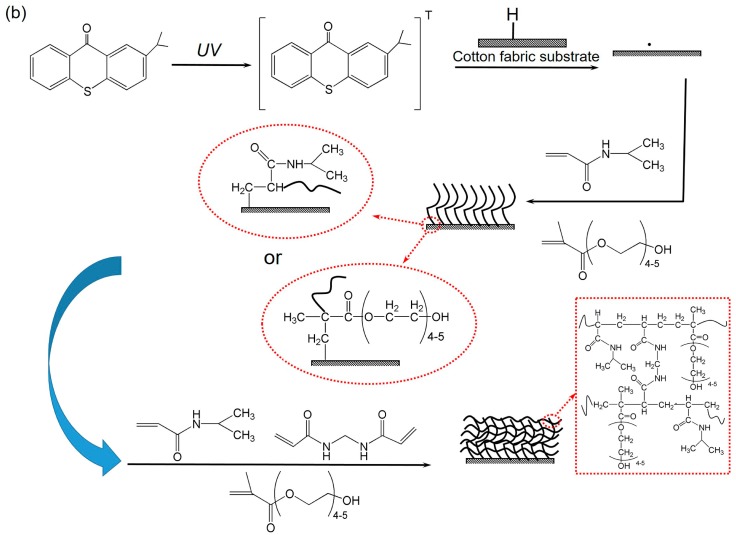
(**a**) UV photo-grafting method for the preparation of fabric grafted hydrogels. (**b**) Schematic illustration of the mechanism of grafting hydrogel on cotton fabrics by UV light.

The UV photo-grafting method was used to prepare cotton fabric grafted hydrogels. In the first step, the cotton fabrics were saturated with acetone solution of 0.1 wt % ITX and exposed to UV light for 8 min to form surface photo-initiators. Then, the pre-treated fabrics were placed on the bottom of the mold, and a proper amount of reaction solution was transferred into the mold, in which the given concentrations of NIPAAm, EGMA_360_, SA, and other additives were dissolved. UV irradiation was carried out in an ultraviolet processor (HWUV400XUV, Zhonghe Machinery Manufacture Co., Ltd., Baoding, China) at room temperature for 30 min. The processor was equipped with a high-pressure mercury lamp (400 W) with a wavelength range of 320–390 nm. After irradiation, the samples were immersed in 1% (*w*/*v*) CaCl_2_ aqueous solution for 24 h and soaked in distilled water to obtain the final purified cotton fabric grafted hydrogels. The procedure and mechanism of UV photo-grafting method is schematically shown in Figure 2.

### 2.4. Characterizations

#### 2.4.1. ATR-FTIR Analysis

ATR-FTIR spectroscopy was used to analyze the chemical structure of these samples. They were dried in a vacuum freeze drier (PD-1C-50, Beijing Boyikang Laboratory Instruments, Beijing, China) and were then measured with a Fourier Infrared Spectrometer (Nicolet 5700, Thermo Nicolet Corporation, Madison, WI, USA) in the range of 4000–400 cm^−1^ and with a resolution of 4 cm^−1^.

#### 2.4.2. Thermogravimetric Analysis

Thermogravimetric analysis of the hydrogel samples was performed with a thermal gravimetric analyzer (TGA) (PYRIS 1, Perkinelmer, Waltham, MA, USA). 10 mg of dried hydrogel samples was used for each measurement at a 20 °C/min heating rate in the range of 30 to 800 °C and with a 50 mL/min nitrogen flow.

#### 2.4.3. DSC Thermal Analysis

A differential scanning calorimeter (Q2000, TA Instruments, New Castle, DE, USA) was used to determine the lower critical solution temperature (LCST) and the glass transition temperature (*T*_g_) of the hydrogel samples. The hydrogel samples were immersed in deionized water at room temperature and allowed to swell for 48 h to reach equilibrium. The swollen samples were then performed from 25 to 40 °C at a 1 °C/min heating rate to measure the LCST, and the dried hydrogel samples were used for the *T*_g_ measurement at 1 °C/min heating rate in the range of −50 °C to 150 °C. The samples for DSC test were around 10 mg, measured in a nitrogen atmosphere.

#### 2.4.4. Surface Morphology

The surface morphology of hydrogel samples was probed by a scanning electron microscope (SEM) (JSM-5610LV, JEOL, Tokyo, Japan). The dried samples were mounted on an aluminum stud using conductive adhesive and sputtered with gold using an auto fine coater (JFC-1600, JEOL, Tokyo, Japan).

#### 2.4.5. Determination of Swelling Behavior

Hydrogel samples were freeze dried at −40 °C for 24 h. The dried samples were weighed before being soaked in distilled water and incubated at 25 °C. The swollen samples were moved out from the water at predetermined time intervals, blot dried, and weighed. This procedure was repeated until the weight of swollen samples reached a constant value. The average values among three measurements were taken for each sample, and the swelling ratio (*SR*) was calculated by Equation (1).

(1) $$SR=(W_{t}-W_{d})/W_{d}$$

where *W_t_* is the mass of the swollen hydrogel at any given time and *W_d_* the mass of the dry hydrogel.

The deswelling kinetics of a hydrogel sample was measured gravimetrically by transferring blot-dried hydrogels that were swollen and reached equilibrium at 25 °C to a water bath of 50 °C. The weight change of the hydrogel was recorded in the course of deswelling at different time intervals. 50 °C is well above the LCST of the hydrogels in this study, and dramatic loss of water could be attained within a short timeframe. All deswelling kinetics studies were carried out in triplicate, and water loss percentage (*WL*%) was defined by Equation (2).

(2) $$WL\%=[(W_{e}-W_{t})/\left(W_{e}-W_{d}\right)]\times 100$$

where *W_e_* is the mass of the swollen hydrogel at equilibrium and the other symbols are the same as defined above.

The temperature-dependent swelling of a hydrogel was characterized by measuring its swelling ratio at 25, 30, 35, 40, 45, 50, 55, and 60 °C. All the experiments were performed in triplicates to get the average value.

#### 2.4.6. Hand Evaluation

The hand evaluation of original cotton fabric and fabric grafted hydrogels were measured by Phabrometer fabric system (Phabromet, Nu Cybertek, Inc., Davis, CA, USA), according to the AATCC Test Method 202. Before being measured, the samples were stored in an atmosphere with a temperature of 20 ± 2 °C and a relative humidity of 65 ± 3% for 24 h. During the measurements, the system monitored the resultant force-displacement curve. The curve area indicates the energy needed to wrinkle the fabrics, which means the fabrics with a smaller curve area has better hand feeling.

## 3. Results and Discussion

### 3.1. ATR-FTIR Analysis of Hydrogels

**Figure 3:**
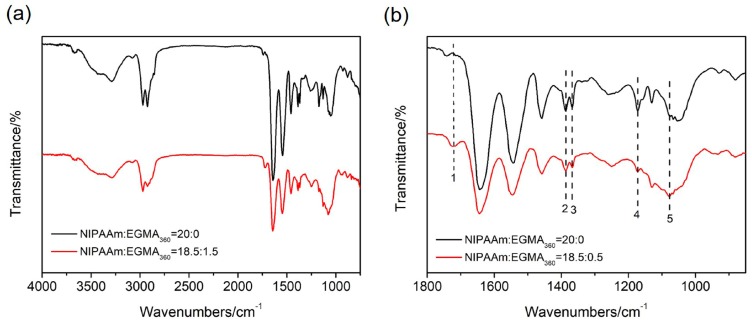
(**a**) ATR-FTIR spectra of alginate-Ca^2+^/P(NIPAAm-*co*-EGMA_360_) hydrogel from 4000 to 600 cm^−1^. The monomer ratio of NIPAAm to EGMA_360_ are 20:0 and 18.5:1.5. (**b**) Zoom-in the ATR-FTIR spectra ranging from 1800 to 850 cm^−1^.

The ATR-FTIR spectra of alginate-Ca^2+^/PNIPAAm hydrogel and alginate-Ca^2+^/P(NIPAAm-*co*-EGMA_360_) hydrogel were shown in Figure 3. As shown in Figure 3a, the broad band in the range of 3600–3300 cm^−1^ (O–H stretching vibrations), 2900–2800 cm^−1^ (C–H stretching vibrations), the amide I band (C=O stretch, ~1643 cm^−1^), and the amide II band (N–H vibration, ~1545 cm^−1^) were identified [28]. When zoom-in the ATR-FTIR spectra in Figure 3b, the small absorbance peaks at 1724 cm^−1^ (guide line 1) was assigned to the C=O stretching vibrations of PEGMA_360_, the absorptions at 1383 cm^−1^ (guide line 2) and 1373 cm^−1^ (guide line 3) were characteristic peaks of PNIPAAm associated with the isopropyl groups, and the absorbance peaks at 1173 cm^−1^ (guide line 4) was identified to the C–N stretching vibrations [28]. The tiny red shift of the C–O stretching absorption band from 1052 cm^−1^ to 1083 cm^−1^ (guide line 5) may have been due to the interactions between the ethoxy groups of PEGMA_360_ and the hydroxyls groups of SA.

### 3.2. Thermogravimetric Analysis (TGA)

**Figure 4:**
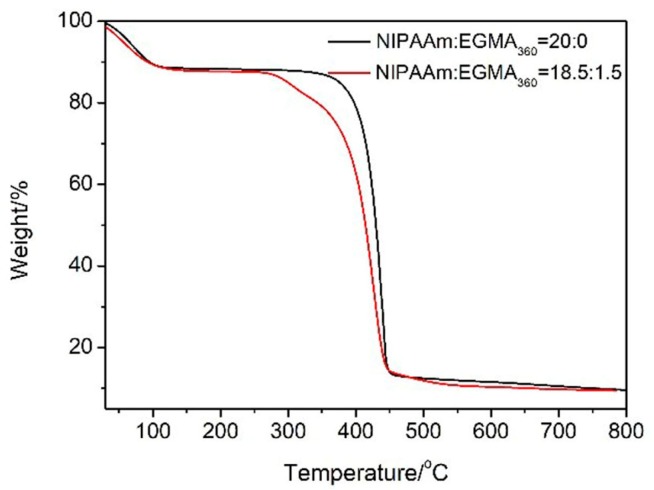
TG curves of alginate-Ca^2+^/P(NIPAAm-*co*-EGMA_360_) hydrogel. The monomer ratios of NIPAAm to EGMA_360_ are 20:0 and 18.5:1.5.

Figure 4 shows the thermogravimetric behavior of alginate-Ca^2+^/P(NIPAAm-*co*-EGMA_360_) hydrogels, which exhibited a multistep mass loss behavior. The weight of the alginate-Ca^2+^/P(NIPAAm-*co*-EGMA_360_) hydrogels underwent a mass loss approximating to 10% below 100 °C, possibly due to the evaporation of free water. At temperature 250–450 °C, the polymer started to decompose by going through a complex process including degradation of side chains and further depolymerization of the main chain structure. The half-weight loss temperatures for the alginate-Ca^2+^/P(NIPAAm-*co*-EGMA_360_) hydrogel (NIPAA:EGMA_360_ = 20:0) and alginate-Ca^2+^/PNIPAAm hydrogel (NIPAA:EGMA_360_ = 18.5:1.5) were 425 °C and 414 °C, respectively. Thus, with an increasing monomer ratio pf EGMA_360_, alginate-Ca^2+^/P(NIPAAm-*co*-EGMA_360_) hydrogels more easily decomposed above 250 °C. However, below 250 °C, the hydrogel with a molar ratio of NIPAAm:EGMA_360_ = 18.5:1.5 still exhibited an excellent thermal stability and had no effect on their application in daily life.

### 3.3. Phase Transition of Hydrogels

**Figure 5:**
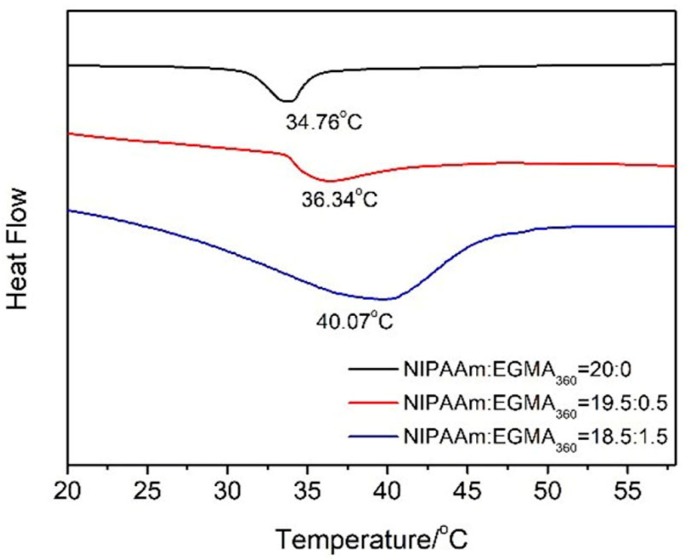
Differential scanning calorimetry (DSC) thermograms of alginate-Ca^2+^/P(NIPAAm-*co*-EGMA_360_) hydrogels. The monomer ratios of NIPAAm to EGMA360 are 20:0, 19.5:0.5 and 18.5:1.5.

The LCST of alginate-Ca^2+^/P(NIPAAm-*co*-EGMA_360_) hydrogels determined from DSC thermogram is shown in Figure 5, where, LCST was defined as the onset temperature of the endothermic peak. The data indicated that the LCST values of alginate-Ca^2+^/P(NIPAAm-*co*-EGMA_360_) hydrogels with monomers ratios of NIPAAm to EGMA_360_ of 20:0, 19.5:1.5 and 18.5:2.5 are 34.76, 36.34 and 40.07 °C, respectively. Thus, the LCSTs of hydrogels shit towards higher value with increase of the EGMA_360_ monomer ratio, which was attributed to the enhanced hydrophilicity. The thermal sensitivity of hydrogels was attributed to their rapid alteration from hydrophilicity to hydrophobicity. Blow the LCST, the hydrophilic groups were bonded to water molecules through hydrogen bonds, and the hydrogels exhibited swollen state in the macroscopic. When the external temperature was increased, the hydrophobic interactions became dominant, leading to shrinkage of the hydrogel [29,30,31]. The introduction of EGMA_360_ into PNIPAAm would increase the hydrophilicity of the hydrogel network due to the hydrophilicity of EGMA_360_. Therefore, the hydrogen bonds between the hydroxyl group in the side chain of EGMA_360_ and the water molecules would be significantly enhanced below the LCST, and higher temperature is required to break these hydrogen bonds.

**Figure 6:**
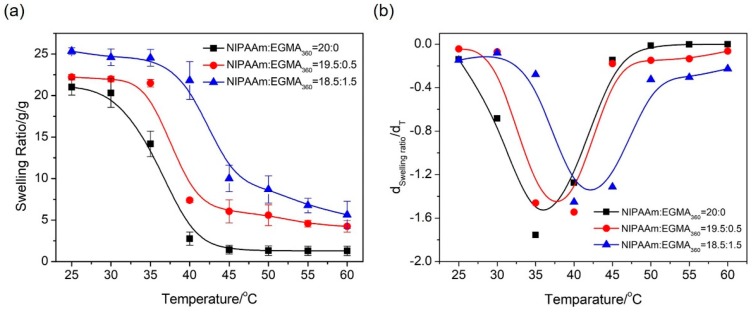
(**a**) Equilibrium swelling ratio of alginate-Ca^2+^/P(NIPAAm-*co*-EGMA_360_) hydrogel as a function of temperature in water. (**b**) First derivatives of the equilibrium swelling ratios with respect to the temperature plotted as a function of temperature. The monomer ratios of NIPAAm to EGMA_360_ are 20:0, 19.5:0.5, and 18.5:1.5.

In order to further investigate the phase transition of alginate-Ca^2+^/P(NIPAAm-*co*-EGMA_360_) hydrogels, the effect of temperature on the water retention capacity of the hydrogels was evaluated by measuring their swelling ratios under external stimuli. The temperature dependent equilibrium swelling ratios in the range from 25 to 60 °C were studied, and the experiment was repeated thrice for each sample and the average values were used in data analysis. As shown in Figure 6a, all the hydrogels presented a thermo-responsive property. The swelling ratio started to decrease when the temperature approached the LCST and was followed by a rapid decrease featuring the phase transition nearby the LCST. The swelling was eventually stabilized when the temperature was above the LCST. The LCST was also determined by plotting the first derivative of the equilibrium swelling ratios with respect to the temperature (Figure 6b). It was clear that LCST shifted toward higher values with the increase of EGMA_360_ ratio, which fit to the result obtained from the DSC thermograms analysis (Figure 5).

### 3.4. Glass Transition Temperature of Hydrogels

**Figure 7:**
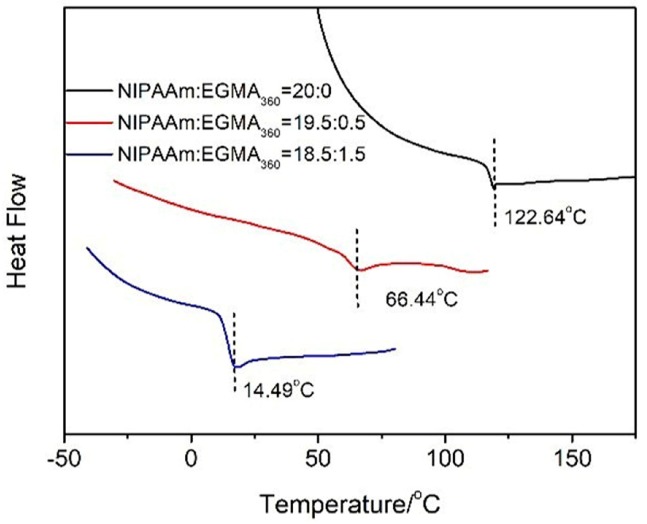
DSC curves of alginate-Ca^2+^/P(NIPAAm-*co*-EGMA_360_) hydrogels. The monomer ratios of NIPAAm to EGMA are 20:0, 19.5:0.5, and 18.5:1.5. *T*_g_ values are indicated by vertical dashed lines.

The influence of the EGMA_360_ ratio to *T*_g_ of the alginate-Ca^2+^/P(NIPAAm-*co*-EGMA_360_) hydrogels was also measured with DSC (Figure 7). The *T*_g_ value of alginate-Ca^2+^/PNIPAAm hydrogel was 122.64 °C and was close to the *T*_g_ of pure PNIPAAm (about 130 °C). The measured *T*_g_s of alginate-Ca^2+^/P(NIPAAm-*co*-EGMA_360_) hydrogels with monomer ratios of 19.5:0.5 and 18.5:1.5 are 66.44 °C and 14.49 °C, respectively. Thus, *T*_g_ is dramatically reduced by increasing the amount of EGMA_360_ in the hydrogels. The EGMA_360_ applied in this study contains 4 or 5 ethoxy groups in the side chain and is regarded as a flexible segment. For this reason, the flexibility of the hydrogel is enhanced after introducing EGMA_360_, which leads to the decrease of *T*_g_.

### 3.5. Swelling of Hydrogels

**Figure 8:**
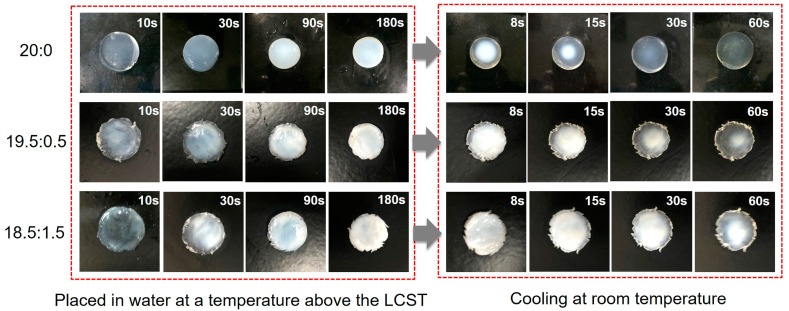
Digital photographs of hydrogels with the change of temperature (the observation time is presented in the upper right of each figure).

The thermal response of hydrogel to external temperatures was captured by a digital camera under a microscope and presented in Figure 8. The hydrogel exhibited a reversible transparent-to-turbid behavior when the external temperature was controlled to cycle around its LCST.

#### 3.5.1. Swelling Kinetics

**Figure 9:**
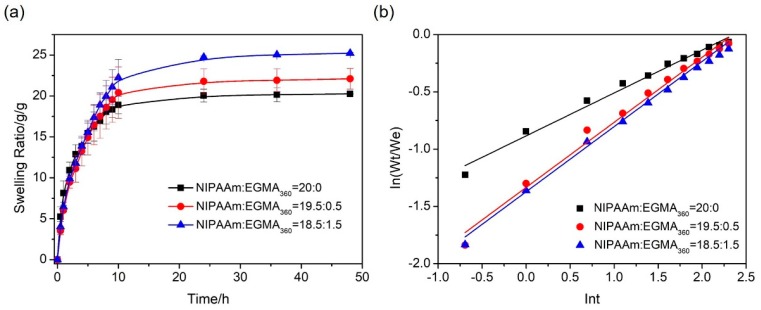
(**a**) Swelling ratio curves and (**b**) ln(*W_t_*/*W_e_*)–lnt curves of alginate-Ca^2+^/P(NIPAAm-*co*-EGMA_360_) hydrogels in water at 25 °C. The monomer ratios of NIPAAm to EGMA_360_ are 20:0, 19.5:0.5, and 18.5:1.5.

The swelling behaviors of hydrogels with different NIPAAm/EGMA_360_ molar ratios were measured by immersing the hydrogels in water at 25 °C for 48 h (Figure 9a). In general, the swelling was fast in the first several h and then gradually slowed down. It reached an equilibrium state in 30–40 h. The swelling capability of hydrogels was enhanced with higher EGMA_360_ contents. For instance, the hydrogel (NIPAAm:EGMA_360_ = 18.5:1.5) possessed the highest swelling ratio (25.22 g/g), while that of alginate-Ca^2+^/PNIPAAm hydrogels (NIPAAm:EGMA_360_ = 20:0) was the lowest (20.25 g/g). The swelling ratios of these hydrogels were mainly related to the hydrophilicity. The additional hydroxyl group at the end of the EGMA_360_ side chain will enhance the hydrophilicity of the hydrogels. For this reason, more PEGMA_360_ component in the hydrogel structure facilitated the hydration and expansion of the hydrogels. In addition, the porous structure of the IPNs greatly improved the diffusion of water into the hydrogel network. Combining these two factors, the swelling capability of the hydrogels with EGMA_360_ is remarkably increased. In our initial experiment, SA and NIPAAm were applied as the monomers, MBAA as chemical cross-linker, APS as initiator, TEMED as catalyst, and CaCl_2_ as physical cross-linker to prepared alginate-Ca^2+^/PNIPAAm IPN hydrogels. Afterward, EGMA_360_ was further introduced into the alginate-Ca^2+^/PNIPAAm networks to form IPN in the later investigation. In order to minimize the effects of the cross-linker to our investigation of the influence of EGMA_360_ to the hydration and transition behaviors, MBAA was still used as the cross-linker in the present investigation. In our future study, we will also choose ethylene glycol dimethacrylate (EGDMA) as the cross-linker to prepare alginate-Ca^2+^/P(NIPAAm-*co*-EGMA) hydrogels, hence the effect of a different cross-linker to the swelling and transition behaviors of hydrogels can be assessed.

In order to understand the swelling kinetics of hydrogels, water diffusion in the hydrogels was analyzed by fitting the swelling data into the power law equation (diffusion/relaxation model) [32,33,34], as shown below.

(3) $$W_{t}/W_{e}=kt^{n}$$

where *W_t_* is the mass of swollen hydrogel at a given time, *W_e_* is the mass of equilibrium swelling hydrogel, *t* is the diffusion time, *k* is a constant, and *n* is the diffusion index. When *n* ≤ 0.5 or *n* ≥ 1, the diffusion kinetics obey Fick or non-Fick diffusion, respectively, whereas the anomalous diffusion refers to the case when 0.5 < *n* <1 [35,36]. Equation (4) can be obtained by taking natural logarithm for both sides of Equation (3) and was used to find the *n* value by determining the slope of the plot ln(*W_t_*/*W_e_*) against ln*t*.

(4) $$\ln(W_{t}/W_{e})=\ln k+n\times \ln t$$

ln(*W_t_*/*W_e_*) as a function of ln*t* was plotted in Figure 9b, it was clear that all three hydrogels presented a linear behavior. Fitting coefficients and characteristic indices extracted from these plots are listed in Table 2. The *n* values are obviously influenced by EGMA_360_ component, which ranged from 0.37, 0.57 to 0.56 with monomer ratios of 20:0, 19.5:0.5, and 18.5:1.5 (NIPAAm:EGMA_360_). It indicated that introduction of EGMA induced the shift of diffusion from a Fickian model to an anomalous model.

#### 3.5.2. Deswelling Kinetics

**Figure 10:**
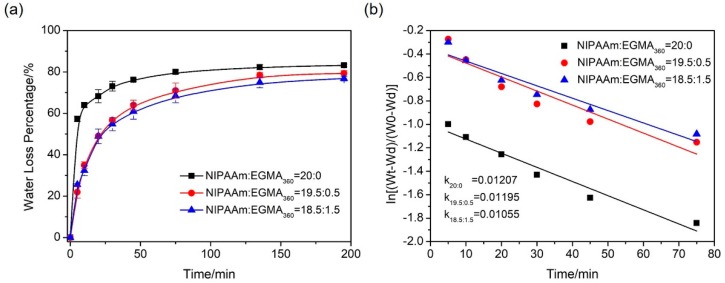
(**a**) Deswelling kinetics of alginate-Ca^2+^/P(NIPAAm-*co*-EGMA_360_) hydrogel in water at 45 °C from the equilibrium swelling state at 25 °C. (**b**) Rate analysis of the deswelling properties of the corresponding hydrogels. The monomer ratios of NIPAAm to EGMA_360_ are 20:0, 19.5:0.5, and 18.5:1.5.

The deswelling kinetics of the hydrogels are shown in Figure 10a. All hydrogel samples exhibited an exponential deswelling behavior with an initially rapid release of water followed by a plateaued water loss percentage starting at around 50 min. As a thermal responsive hydrogel was transferred from the water bath of 25 °C to that of 50 °C, the internal pressure started to build up and drive the water to flow out. The internal pressure gradually reduced as a result of water outflow until the system reached another pressure-volume balance. As seen in Figure 10a, the alginate-Ca^2+^/PNIPAAm hydrogels shrank faster than did the alginate-Ca^2+^/P(NIPAAm-*co*-EGMA_360_) hydrogels by losing a larger volume of water in same time frame. Being kept in 50 °C water for about 20 min, the alginate-Ca^2+^/PNIPAAm hydrogels shrank and lost 68% water, while alginate-Ca^2+^/P(NIPAAm-*co*-EGMA_360_) hydrogels still held about 50% water. On the other hand, the ultimate water loss percentage of the alginate-Ca^2+^/P(NIPAAm-*co*-EGMA_360_) hydrogels slightly decreases with the increase of EGMA_360_ ratio. It is well known that when the temperature is above the LCST, the thermo-responsive polymer switches from the hydrophilic state to hydrophobic state. Thus, the shrinkage of the hydrogels sets in. For the IPN hydrogels, EGMA_360_ was introduced into the alginate-Ca^2+^/PNIPAAm networks to form IPN, the hydrophilcity of the hydroxyl groups at the side chain of PEGMA_360_ is better than that of acylamino groups in PNIPAAm, resulting in relative difficultly removing of water molecules from the gel matrix. Thus, the shrinking rates were slightly weakened.

To quantitatively analyze the deswelling behavior, a semi-logarithmic plot as a first ordered rate analysis was applied to the time dependence deswelling kinetics analysis [37,38].

(5) $$\ln\left[\left(W_{t}-W_{d}\right)/\left(W_{0}-W_{d}\right)\right]=-kt$$

where *k* is the shrinkage constant, and a greater *k* means a faster shrinkage process [21].

Figure 10b showed the time dependence of $\ln\left[\left(W_{t}-W_{d}\right)/\left(W_{0}-W_{d}\right)\right]$. It presented a linear behavior. The shrinkage constants were obtained from the slopes of the linear fitting. The hydrogel (NIPAAm:EGMA_360_ = 20:0) provides the largest shrinkage rate constant (*k* = 0.01207), followed by the one with NIPAAm:EGMA_360_ = 19.5:0.5 (*k* = 0.01195) and 18.5:1.5 (*k* = 0.01055). The smaller value of *k* is an indicator of lower release rate, which agrees with the results of deswelling kinetic curves in Figure 10a.

### 3.6. ATR-FTIR Analysis of Cotton Fabric Grafted Hydrogels

**Figure 11:**
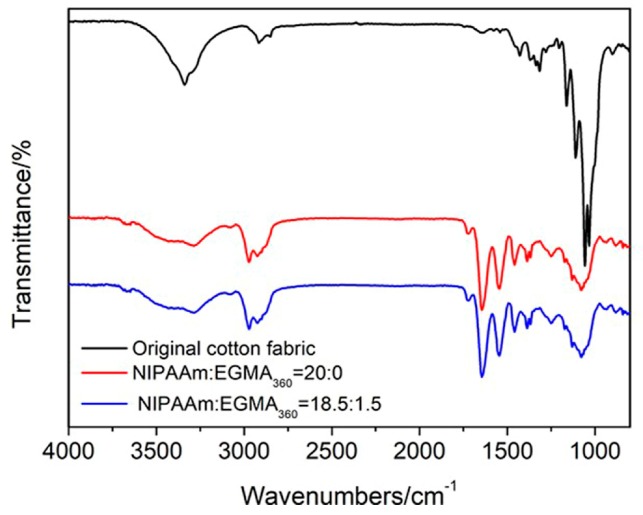
(**a**) ATR-FTIR spectra of original cotton fabric and cotton fabric grafted alginate-Ca^2+^/P(NIPAAm-*co*-EGMA_360_) hydrogel from 4000 to 600 cm^−1^. The monomer ratio of NIPAAm to EGMA_360_ are 20:0 and 18.5:1.5. (**b**) Zoom-in the ATR-FTIR spectra ranging from 1800 to 850 cm^−1^.

Immobilization of alginate-Ca^2+^/P(NIPAAm-*co*-EGMA_360_) IPN hydrogel on cotton fabrics by photo-grafting was confirmed by ATR-FTIR measurements (Figure 11a). The black curve presented the typical ATR-FTIR spectrum of the original cotton fabrics, including the main absorption peaks, such as O–H stretching vibrations (3600–3300 cm^−1^), C–H stretching vibrations (3000–2800 cm^−1^), and C–O stretching vibrations (1057 cm^−1^). After grafting alginate-Ca^2+^/P(NIPAAm-*co*-EGMA_360_) IPN hydrogel to the cotton fabrics, all the characteristic peaks (such as C=O, N–H, isopropyl groups, etc.) observed in alginate-Ca^2+^/P(NIPAAm-*co*-EGMA_360_) IPN hydrogel spectra (Figure 3) are all observed in the red and blue curves. It indicates the successful immobilization of alginate-Ca^2+^/P(NIPAAm-*co*-EGMA_360_) IPN hydrogel on the surface of cotton fabrics.

### 3.7. Morphological Observation

**Figure 12:**
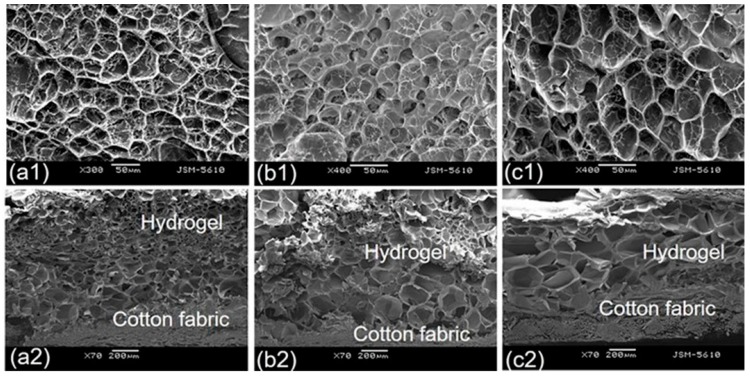
Scanning electron microscopy (SEM) images of the top view (**a1**–**c1**) and the cross section (**a2**–**c2**) of cotton fabric grafted with alginate-Ca^2+^/P(NIPAAm-*co*-EGMA_360_) hydrogels. The monomer ratios of NIPAAm to EGMA_360_ are (**a1**,**a2**) 20:0, (**b1**,**b2**) 19.5:0.5, and (**c1**,**c2**) 18.5:1.5.

The morphology of cotton fabric–grafted alginate-Ca^2+^/P(NIPAAm-*co*-EGMA_360_) hydrogels in swollen condition (swollen hydrogels were freeze-dried before SEM examination) is shown in Figure 12. The structure of the samples may slightly collapse due to the freeze-drying treatment, but dramatic morphological differences could be observed among all hydrogels.

The data clearly illustrated the dependence of hydrogel morphology on the ratio of NIPAAm to EGMA_360_. The hydrogels exhibited numerous interconnected pore structure. Moreover the pore of these honeycomb-like structures were enlarged with an increase of EGMA_360_ content in the IPN hydrogel composition, i.e., the hydrogels of NIPAAm:EGMA_360_ = 18.5:1.5 had the largest pore size, while that of NIPAAm:EGMA_360_ = 20:0 had the smallest. The results may be attributed to the highly expanded network formed during the crosslinking polymerization process due to the electrostatic repulsions among the hydroxyl (–O^−^) from EGMA_360_ and the carboxylates (–COO^−^) from SA [39]. The introduction of EGMA_360_ to hydrogel influenced the crosslinking density as well as the hydrophilic/hydrophobic balance of alginate-Ca^2+^/PNIPAAm hydrogels. Hence, the microstructure of hydrogels experienced a significant change.

### 3.8. Temperature Dependence of Fabric Grafted Hydrogels

**Figure 13:**
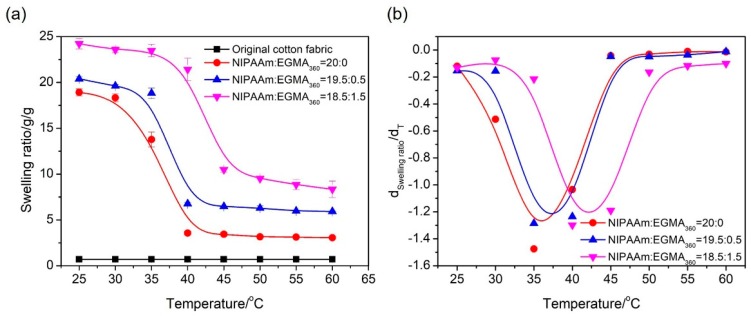
(**a**) Equilibrium swelling ratio of cotton fabric grafted alginate-Ca^2+^/P(NIPAAm-*co*-EGMA_360_) hydrogels as a function of temperature in water. (**b**) First derivatives of the equilibrium swelling ratios with respect to the temperature plotted as a function of temperature. The monomer ratios of NIPAAm to EGMA_360_ are 20:0, 19.5:0.5, and 18.5:1.5.

The thermo-responsive property of the fabric grafted hydrogels was analyzed by measuring their swelling ratios in different temperatures (Figure 13). There was no change in swelling ratio for the original cotton fabric upon heating, indicating the original cotton was not thermo-responsive. However, the fabric grafted alginate-Ca^2+^/P(NIPAAm-*co*-EGMA_360_) hydrogels presented a typical sudden shrinkage of swelling ratio at 35.58, 36.94 and 42.43 °C (Figure 13b), which is close to the LCST of alginate-Ca^2+^/P(NIPAAm-*co*-EGMA_360_) hydrogels. This phenomenon indicates that the incorporation of cotton fabrics has little influence on the thermal transition of hydrogels.

### 3.9. Hand Feeling of Cotton Fabric Grafted Hydrogels

**Figure 14:**
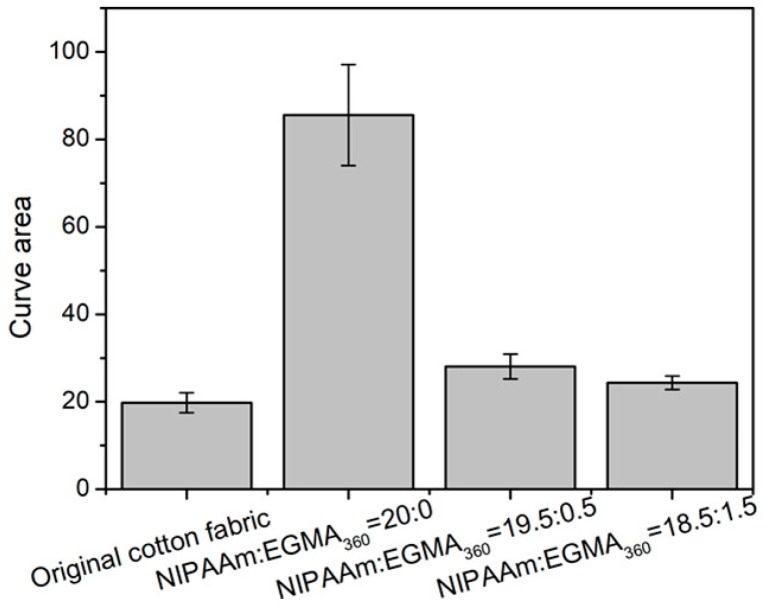
Curve areas of the cotton fabric grafted with alginate-Ca^2+^/P(NIPAAm-*co*-EGMA_360_) hydrogels. The monomer ratios of NIPAAm to EGMA_360_ are 20:0 19.5:0.5, and 18.5:1.5.

Extraction curve area indicates the energy needed to wrinkle the fabrics, which can be obtained from Phabrometer Fabric System. As the value is inversely proportional to hand values, it can be used to evaluate the hand feeling of the fabrics. Generally, the smaller the curve area, the better the hand feeling of the fabric. Figure 14 showed all the test data of curve areas, it was clearly that the curve area of the original cotton fabric (19.76) and the cotton fabric grafted with alginate-Ca^2+^/P(NIPAAm-*co*-EGMA_360_) hydrogels with NIPAAm:EGMA_360_ = 19.5:0.5 (28.07) and 18.5:1.5 (24.38) exhibit no prominent difference. Hence, it can be concluded that the grafted alginate-Ca^2+^/P(NIPAAm-*co*-EGMA_360_) hydrogels do not affect the hand feeling of the cotton fabrics. On the contrary, the cotton fabrics with alginate-Ca^2+^/PNIPAAm hydrogels shows a much larger value (85.57). The huge difference among these curve areas would be attributed to the decrease of *T*_g_ value (the measured *T*_g_ is ranged from 122.64 °C, 66.44 °C to 14.49 °C with monomer ratio of NIPAAm:EGMA_360_ changed from 20:0, 19.5:0.5, and 18.5:1.5). As the flexibility of hydrogel is enhanced when the *T*_g_ is decreased, the hand feeling of the fabrics with hydrogel is also improved.

## 4. Conclusions

In the present work, the influence of ethylene glycol methacrylate (EGMA_360_) to the hydration and transition behaviors of a thermo-responsive interpenetrating network (IPN) hydrogels series consisting of sodium alginate, *N*-isopropylacrylamide (NIPAAm) and ethylene glycol methacrylate (EGMA_360_) were investigated. Polymerization of the resulting IPN hydrogels was confirmed by ATR-FTIR and TGA analyses. DSC and temperature stimulus-responsive equilibrium swelling ratio results indicated that hydrogels with increased EGMA_360_ composition ratio exhibit higher LCST. Additionally, *T*_g_ was dramatically shifted towards lower temperatures due the flexible ethoxy groups in EGMA_360_. Besides, the swelling/deswelling behaviors indicated that a faster water uptake rate and a slower water loss rate appeared with increase of EGMA_360_ amount. The obtained IPN hydrogels were grafted to the cotton fabric by UV photo-grafting method. SEM was utilized to investigate the morphology and the results demonstrated the porous structure of hydrogel layers. Moreover, the pores of the hydrogels were enlarged with larger amount of EGMA_360_. Further, the equilibrium swelling ratio of the fabric grafted hydrogels in water decreased upon heating, which confirmed the thermo-responsive property of fabrics with hydrogels. Unlike the fabrics grafted with alginate-Ca^2+^/PNIPAAm hydrogel, the cotton fabric grafted alginate-Ca^2+^/P(NIPAAm-*co*-EGMA_360_) hydrogels present similar hand feeling to the original cotton fabrics. Therefore, alginate-Ca^2+^/P(NIPAAm-*co*-EGMA_360_) hydrogel is a promising candidate for applications in the field of biomedical textiles, such as the wound dressings.

## Figures and Tables

**Table 1:** Conditions for preparation of hydrogels.

Sample Code	SA/g	NIPAAm/EGMA_360_/mol/mol	MBAA/NIPAAm/%(mol/mol)	APS/mg	TEMED/μL	DI Water/mL
1	0.2	20:0	1.0	20	6	20
2	0.2	19.5:0.5	1.0	20	6	20
3	0.2	18.5:1.5	1.0	20	6	20

**Table 2:** Swelling kinetic parameters of alginate-Ca^2+^/P(NIPAAm-*co*-EGMA_360_) hydrogels.

Sample	Characteristic Index (*n*)	Fitting Coefficient
NIPAAm:EGMA_360_ = 20:0	0.37	0.99
NIPAAm:EGMA_360_ = 19.5:0.5	0.57	0.99
NIPAAm:EGMA_360_ = 18.5:1.5	0.56	0.99
